# Supplementary material for: Broadly neutralizing antibodies target a haemagglutinin anchor epitope
Source: Nature. 2021 Dec 23;602(7896):314–20. doi: 10.1038/s41586-021-04356-8 (PMC8828479; doi:10.1038/s41586-021-04356-8)
Supplement: Supplementary file 1 — This file contains Supplementary Fig. 1 and Supplementary Tables 1–6. [file 41586_2021_4356_MOESM1_ESM.pdf]

nature portfolio

https://doi.org/10.1038/s41586-021-04356-8

Supplementary information

Broadly neutralizing antibodies target a haemagglutinin anchor epitope

In the format provided by the authors and unedited

Broadly neutralizing antibodies target a hemagglutinin anchor epitope

Authors: Jenna J. Guthmiller[1,18]*, Julianna Han[2,18], Henry A. Utset[1], Lei Li[1], Linda Yu-Ling Lan[3], Carole Henry[1,16], Christopher T. Stamper[3], Meagan McMahon[4], George O'Dell[4], Monica L. Fernández-Quintero[5], Alec W. Freyn[4,16], Fatima Amanat[4], Olivia Stovicek[1], Lauren Gentles[6,7], Sara T. Richey[2], Alba Torrents de la Peña[2], Victoria Rosado[4], Haley L. Dugan[3], Nai-Ying Zheng[1], Micah E. Tepora[1], Dalia J. Bitar[1], Siriruk Changrob[1], Shirin Strohmeier[4], Min Huang[1], Adolfo García-Sastre[4,8,9,10,11], Klaus R. Liedl[5], Jesse D. Bloom[6,7,12,13], Raffael Nachbagauer[4,16], Peter Palese[4,8], Florian Krammer[4], Lynda Coughlan[14,15], Andrew B. Ward[2]*, Patrick C. Wilson[1,3,17]*

Affiliations:

[1]Department of Medicine, Section of Rheumatology, University of Chicago, Chicago, IL 60637, USA

[2]Department of Integrative Structural and Computational Biology, The Scripps Research Institute, La Jolla, CA 92037, USA

[3]Committee on Immunology, University of Chicago, Chicago, IL 60637, USA

[4]Department of Microbiology, Icahn School of Medicine at Mount Sinai, New York, NY 10029, USA

[5]Center for Molecular Biosciences Innsbruck, Department of General, Inorganic and Theoretical Chemistry, University of Innsbruck, Innsbruck, Austria

[6]Basic Sciences Division, Fred Hutchinson Cancer Research Center, Seattle, WA 98109, USA

[7]Department of Microbiology, University of Washington, Seattle, WA 98195, USA

[8]Department of Medicine, Division of Infectious Diseases, Icahn School of Medicine at Mount Sinai, New York, NY 10029, USA

[9]Global Health and Emerging Pathogens Institute, Icahn School of Medicine at Mount Sinai, New York, NY 10029, USA

[10]The Tisch Cancer Center, Icahn School of Medicine at Mount Sinai, New York, NY 10029, USA

[11]Department of Pathology, Molecular and Cell-Based Medicine, Icahn School of Medicine at Mount Sinai, New York, NY 10029, USA

[12]Department of Genome Sciences, University of Washington, Seattle, WA 98195, USA

[13]Howard Hughes Medical Institute, Fred Hutchinson Cancer Research Center, Seattle, WA 98109, USA

[14]Department of Microbiology and Immunology, University of Maryland School of Medicine, Baltimore, MD 21201, USA

[15]Center for Vaccine Development and Global Health (CVD), University of Maryland School of Medicine, Baltimore, MD 21201, USA

[16]Present address: Moderna Inc., Cambridge, MA 02139, USA

[17]Present address: Drukier Institute for Children's Health and Department of Pediatrics, Weill Cornell Medicine, New York, NY 10021

[18]These authors contributed equally

*Correspondence: jguthmiller@uchicago.edu (J.J.G.); andrew@scripps.edu (A.B.W.); pcw4001@med.cornell.edu (P.C.W.)

Table of Contents

1. **Supplementary Figures**
    1. Supplementary Fig. 1: Gating Strategy for Extended Data Fig. 6h. (Page 4)
2. **Supplementary Tables**
    1. Supplementary Table 1: mAbs used in cocktails for *in vivo* studies and neutralization potency against A/Netherlands/602/2009. (Page 5)
    2. Supplementary Table 2: HA and 222-1C06 Fab interactions (Page 6)
    3. Supplementary Table 3: Fab:Fab interactions of 222-1C06 (Page 7)
    4. Supplementary Table 4: Reference H1 strains used in Fig. 2j. (Page 8)
    5. Supplementary Table 5: Reference Group 1 HA strains used in Fig. 2k. (Page 9)
    6. Supplementary Table 6: Accession numbers for anchor-binding mAbs. (Page 10)

Supplemental Fig. 1

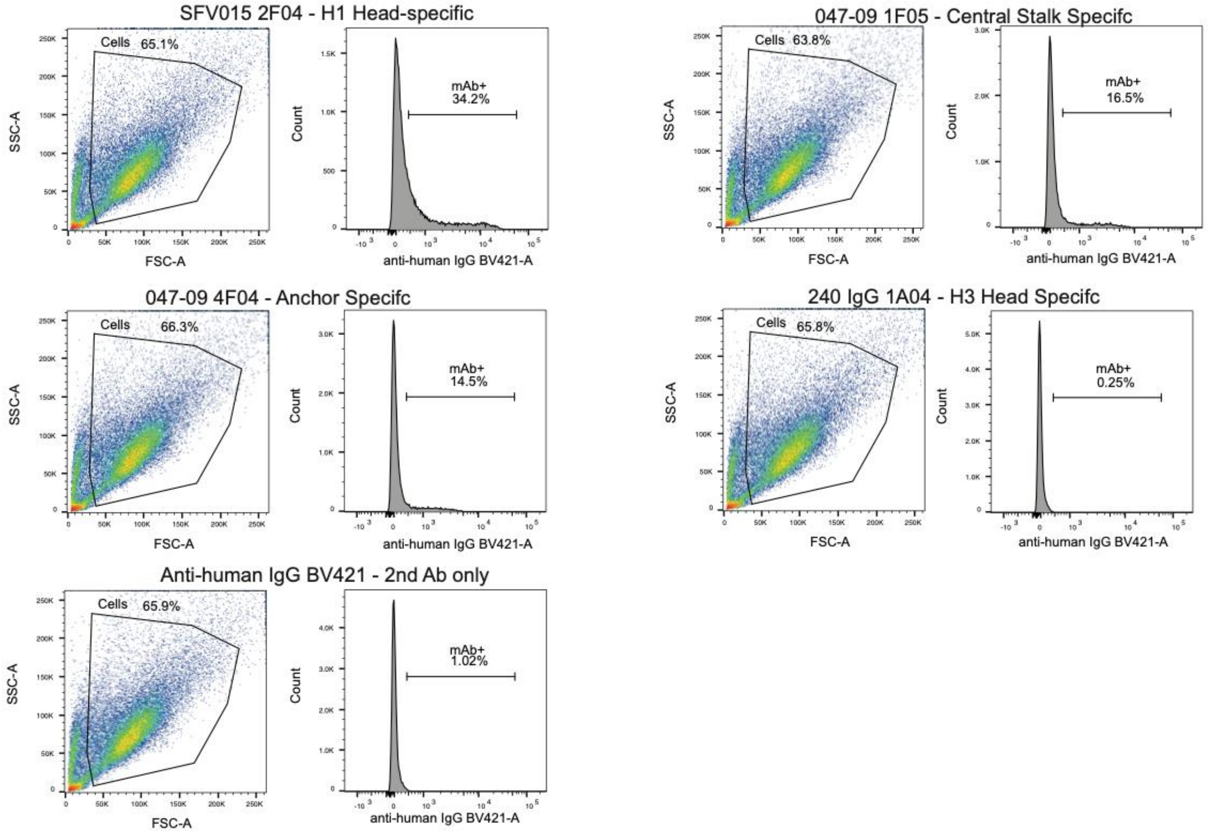

Supplementary Fig. 1: Gating Strategy for Extended Data Fig. 6h. Examples of gating for total cells and mAb$^+$ cells in samples that were transfected to express membrane-bound A/California/7/2009 HA relating gMFI presented in Extended Data Fig. 6h.

Supplementary Table 1: mAbs used in cocktails for *in vivo* studies and neutralization potency against A/Netherlands/602/2009.

mAb name	Epitope Specificity	A/Netherlands/602/2009 IC_{50} (μg/ml)
241 IgG 2A06	Anchor	22.99
047-09 4F04	Anchor	66.63
030-09 3E05	Anchor	39.71
SFV009 3G01	Anchor	7.588
236 IgG 1A02	Anchor	66.88
045-09 2B06	Central Stalk	55.03
SFV005 2G02	Central Stalk	9.342
SFV019 4E03	Central Stalk	7.221
220 IgG 1A05	Central Stalk	9.536
241 IgA 2E06	Central Stalk	10.40

Supplementary Table 2: HA and 222-1C06 Fab interactions.

HA Residues	Fab
S361	K-CDR3 N93
Q356	K-CDR3-W94<br>H-CDR3 W99<br>H-CDR2 Y58
E358 backbone	H-CDR2 Y58
Aromatic Pocket:<br>W343<br>H354<br>Y363	Aromatic Pocket:<br>H-CDR3 W99<br>H-CDR3 P100<br>K-CDR3 W94
backbone positions 344-345	K-CDR1 R30

Supplementary Table 3: Fab: Fab interactions of 222-1C06.

**Fab:Fab interactions**
Intra-fab aromatic pocket
K-CDR3 W94, P95, P96 –<br>Heavy Chain W47, Y50, Y59, W99, Y100c
K-CDR3 W94 – K-CDR3 P95
K-CDR3 P95 – HCDR2 Y59
K-CDR3 W94 – H-CDR3 Y100c
K-CDR3 P96 – H-FR2 W47

Supplementary Table 4: Reference H1 strains used in Fig. 2j.

Subtype	Strain Name
H1N1	A/Quebec/25/2013
H1N1	A/Czech Republic/4/2016
H1N1	A/Helsinki/77/2013
H1N1	A/New York/18/2009
H1N1	A/swine/Mexico/AVX27/2012
H1N2	A/swine/Korea/PZ4/2006
H1N2	A/swine/Ohio/8910/2001
H1N1	A/swine/North Carolina/SG1279/2007
H1N1	A/swine/Minnesota/00991/2006
H1N1	A/swine/Hong Kong/1937/1994
H1N1	A/swine/Wisconsin/1915/1988
H1N1	A/swine/Wisconsin/11/1976
H1N2	A/swine/Miyagi/5/2003
H1N1	A/swine/Manitoba/D0270/2013
H1N1	A/swine/Ohio/23/1935
H1N1	A/swine/USA/1976/1931
H1N1	A/South Carolina/1/1918
H1N1	A/Iran/1417/2016
H1N1	A/Henry/1936
H1N1	A/Albany/1618/1951
H1N1	A/Roma/1949
H1N2	A/swine/England/463180/1994
H1N2	A/swine/Italy/233139/2005
H1N2	A/swine/England/448813/1994
H1N2	A/swine/England/063782/2013
H1N1	A/Singapore/6/1986
H1N1	A/Ostrova/801/1998
H1N2	A/swine/Minnesota/A01201102/2011
H1N2	A/swine/Minnesota/A01567011/2014
H1N2	A/swine/North Carolina/2858/2010
H1N1	A/swine/IL/00685/2005
H1N1	A/Auckland/585/2000
H1N1	A/California/UR060479/2007
H1N3	A/pintail/Aomori/1130/2008
H1N3	A/duck/Fujian/FJ1239/2014
H1N1	A/duck/Bavaria/1/1977
H1N9	A/thickbilled murre/Alaska/390/1976
H1N9	A/northern shoveler/California/K168/2005
H1N1	A/yellow-billed pintail/Chile/1/2012
H1N1	A/mallard duck/Chile/C4079/2015
H1N1	A/swine/Arnsberg/6554/1979
H1N1	A/swine/Germany/8533/1991
H1N2	A/swine/Italy/195369/2010
H1N1	A/swine/Hong Kong/1559/2008
H1N1	A/swine/Cotes d’Armor/1624/2002
H1N1	A/swine/Jiangsu/J004/2018

Supplementary Table 5: Reference Group 1 HA strains used in Fig. 2k.

HA Subtype	Strain Name
H1	A/California/04/2009
H2	A/Singapore/1/1957
H5	A/mallard/Italy/3401/2005
H6	A/chicken/Taiwan/0705/1999
H8	A/turkey/Ontario/6118/1968
H9	A/swine/Hong Kong/9/1998
H11	A/duck/England/1/1956
H12	A/duck/Alberta/60/1976
H13	A/gull/Maryland/704/1977
H16	A/black-headed-gull/Turkmenistan/13/1976
H17	A/little-yellow-shouldered-bat/Guatemala/060/2010
H18	A/flat-faced bat/Peru/033/2010

Supplementary Table 6: Accession numbers for anchor-binding mAbs.

mAb Name	Heavy Chain	Light Chain
045-09 1A03	KM604153.1	KM604154.1
045-09 2B03	KU167232.1	KU167360.1
047-09 4F04	KU167249.1	KU167375.1
030-09 3E05	KU167198.1	KU167333.1
SFV009 3D04	JX027398.1	JX027386.1
SFV009 3G01	JX027395.1	KM604174.1
SFV009 3G03	JX027404.1	JX027389.1
030-09M_1B06	OK666530.1	OK666527.1
121-2C06	OK666531.1	OK666528.1
222-1C06	OK666532.1	OK666529.1
241-14 IgG 2A06	MW079606.1	MW079713.1
241-14 IgA 1D05	MW079655.1	MW079762.1
241-14 IgA 2F04	MW079694.1	MW079801.1
241-14 IgA 2F06	MW079695.1	MW079802.1
236-14 IgG 1A02	MW079617.1	MW079725.1
236-14 IgG 1D01	MW079618.1	MW079726.1
236-14 IgG 1F01	MW079624.1	MW079732.1
236-14 IgA 1F06	MW079667.1	MW079776.1
