# Supplementary material for: Broadly neutralizing antibodies target a haemagglutinin anchor epitope
Source: Nature. 2021 Dec 23;602(7896):314–20. doi: 10.1038/s41586-021-04356-8 (PMC8828479; doi:10.1038/s41586-021-04356-8)
Supplement: Supplementary file 2 — Reporting Summary [file 41586_2021_4356_MOESM2_ESM.pdf]

nature portfolio

Corresponding author(s): Jenna Guthmiller, Andrew Ward, Patrick Wilson

Last updated by author(s): Dec 3, 2021

Reporting Summary

Nature Portfolio wishes to improve the reproducibility of the work that we publish. This form provides structure for consistency and transparency in reporting. For further information on Nature Portfolio policies, see our Editorial Policies and the Editorial Policy Checklist.

Statistics

For all statistical analyses, confirm that the following items are present in the figure legend, table legend, main text, or Methods section.

n/a	Confirmed	
☐	☒	The exact sample size (*n*) for each experimental group/condition, given as a discrete number and unit of measurement
☐	☒	A statement on whether measurements were taken from distinct samples or whether the same sample was measured repeatedly
☐	☒	The statistical test(s) used AND whether they are one- or two-sided<br>*Only common tests should be described solely by name; describe more complex techniques in the Methods section.*
☒	☐	A description of all covariates tested
☒	☐	A description of any assumptions or corrections, such as tests of normality and adjustment for multiple comparisons
☐	☒	A full description of the statistical parameters including central tendency (e.g. means) or other basic estimates (e.g. regression coefficient) AND variation (e.g. standard deviation) or associated estimates of uncertainty (e.g. confidence intervals)
☐	☒	For null hypothesis testing, the test statistic (e.g. *F*, *t*, *r*) with confidence intervals, effect sizes, degrees of freedom and *P* value noted<br>*Give P values as exact values whenever suitable.*
☒	☐	For Bayesian analysis, information on the choice of priors and Markov chain Monte Carlo settings
☒	☐	For hierarchical and complex designs, identification of the appropriate level for tests and full reporting of outcomes
☒	☐	Estimates of effect sizes (e.g. Cohen's *d*, Pearson's *r*), indicating how they were calculated

Our web collection on statistics for biologists contains articles on many of the points above.

Software and code

Policy information about availability of computer code

Data collection	Software - Leginon beta and v3.2, Appion beta
Data analysis	Code - dms_tools2 version 2.4.12, dms_tools version 1.1.12<br>Software - Prism 8, Prism 9, VGene (beta), Librator (beta), Cellrander Single-Cell Software suite v3.0, Seurat 3 v3.2.0, LinQ-View v0.99, WebLogo v2.8.2, UCSF Chimera 1.14, Pymol v2.3.4, Rosettaantibody v2021, MOE v2020, AMBER v20, GROMACS 2019.2 with plumed-2.5.2., PyEMMA v2.5.7, FlowJo v10, MotionCor v2, Relion v3.0, XQuartz v2.7.11, abYsis v3.4.1, Rosetta 2020.03.61102, coot 0.9-pre EL, CryoSPARC2 3.2.0, Phenix 1.17.1-3660, Clustal Omega (https://www.ebi.ac.uk/Tools/msa/clustalo/).

For manuscripts utilizing custom algorithms or software that are central to the research but not yet described in published literature, software must be made available to editors and reviewers. We strongly encourage code deposition in a community repository (e.g. GitHub). See the Nature Portfolio guidelines for submitting code & software for further information.

Data

Policy information about availability of data

All manuscripts must include a data availability statement. This statement should provide the following information, where applicable:

- Accession codes, unique identifiers, or web links for publicly available datasets
- A description of any restrictions on data availability
- For clinical datasets or third party data, please ensure that the statement adheres to our policy

Repertoire data generated from single cell RNA-sequencing data is deposited at Mendeley Data (https://data.mendeley.com/datasets/jzsx489pmk/1). Accession numbers for all other anchor-targeting mAbs are included in Supplemental Table 1. Electron microscopy maps were deposited to the Electron Microscopy DataBank under accession IDs: D_100025433, D_1000254374, D_1000254375, D_1000254376, D_1000254377, D_1000254378, D_1000254383, D_1000254384,

D_1000254385, D_1000254386, D_1000254388, D_1000254379, D_1000254391, and D_1000254382. All next generation sequencing data for 045-09 2B06 deep mutational scanning and for the H1N1 mutational scanning can be found on the Sequence Read Archive under BioProject accession number PRJNA309339. The following Protein Database accession numbers were downloaded and included in the manuscript - 6HJQ, 3SDY, 4NM8, 6HJQ, 4M4Y, 4WE4, 4JTV, 4FQI, 3ZTN, and 7MEM.

Field-specific reporting

Please select the one below that is the best fit for your research. If you are not sure, read the appropriate sections before making your selection.

☒ Life sciences ☐ Behavioural & social sciences ☐ Ecological, evolutionary & environmental sciences

For a reference copy of the document with all sections, see nature.com/documents/nr-reporting-summary-flat.pdf

Life sciences study design

All studies must disclose on these points even when the disclosure is negative.

Sample size	Samples sizes were based on the number of donors and ability to process samples.
Data exclusions	No data were excluded.
Replication	All experiments were performed more than once, except the mouse viral titers. All replications were successful.
Randomization	Recipients for the cHA vaccine arms were randomized. Otherwise, no other experimental groups were used and therefore were not randomized.
Blinding	Serum competition ELISAs in Fig. 4 and Extended Data 6 were blinded. Otherwise, no other blinding was performed.

Reporting for specific materials, systems and methods

We require information from authors about some types of materials, experimental systems and methods used in many studies. Here, indicate whether each material, system or method listed is relevant to your study. If you are not sure if a list item applies to your research, read the appropriate section before selecting a response.

Materials & experimental systems

n/a	Involved in the study	
☐	☒	Antibodies
☐	☒	Eukaryotic cell lines
☒	☐	Palaeontology and archaeology
☐	☒	Animals and other organisms
☐	☒	Human research participants
☐	☒	Clinical data
☒	☐	Dual use research of concern

Methods

n/a	Involved in the study	
☒	☐	ChIP-seq
☐	☒	Flow cytometry
☒	☐	MRI-based neuroimaging

Antibodies

Antibodies used

Anti-human IgG Fc - BV421, clone M1310G05, Biolegend, Cat#410703
Mouse anti-influenza A nucleoprotein antibody, clone A3, biotin, Sigma/Millipore, MAB8258B-5
Polyclonal Goat Anti-human IgG HRP, Jackson Immunoresearch, Cat#109-035-098
Polyclonal Goat anti-mouse IgG HRP, Southern Biotech, Cat# 1030-05
Polyclonal Goat anti-mouse IgG H&L peroxidase-conjugated, Rockland Cat#610-1302

Monoclonal antibodies from previous publications and synthesized in-house are listed below with reference -
CR9114 - 10.1126/science.1222908
3H9 - 10.1073/pnas.84.24.9150

Monoclonal antibodies unique to this study and synthesized in-house are listed below -
030-09 3E05
030-09M 1B06
045-09 1A03
045-09 2B03
047-09 4F04
SFV009 3D04
SFV009 3G01
SFV009 3G03
236 IgG 1A02

236 IgG 1D01
236 IgG 1F01
236 IgA 1F06
241 IgG 2A06
241 IgA 1D05
241 IgA 2F04
241 IgA 2F06
121 2C06
222 1C06
301_91
301_249
301_275
308_60
310_10
310_49
317_30
317_117
319_75
319_147
319_345
319_373
322_48
324_5
326_38
326_42
326_50
327_32
334_52
334_53
334_62
337_32
337_43
337_95
346_54
347_64
347_246
350_8
350_22
350_132
350_145
351_38
351_93
351_139
029-09 4A01
047-09 1F05
047-09 4E01
051-094B02
051-09 4C06
051-09 4E06
051-09 5A02
051-09 5C01
030-09 1A06
030-09 1E04
030-09 2B03
030-09 2G03
030-09 3B03
045-09 2B06
SFV005 2G02
SFV019 4E03
SFV009 2A06
SFV009 3A01
sc1009 3B05
sc1009 3E06
SF1000 3D04
sc70 1F02
sc70 5B03
039-10 5F06
039-10 5G02
039-10 5G05
240 IgG 1C04
220 IgG 1A05
237 IgG 1D01
241 IgA 1D05
241 IgA 2E06
301-48
301-90

	301-277<br>308-2<br>311-30<br>319-4<br>319-73<br>319-99<br>319-256<br>319-418<br>324-184<br>334-98<br>337-51<br>337-53<br>347-58<br>347-140<br>351-31
Validation	All commercial antibodies were validated by their manufacturers and were titrated in the lab to determine optimal concentration for experimentation. In-house generated monoclonal antibodies were validated in preliminary ELISAs to A/California/7/2009 H1N1 virus or recombinant H1 or cH5/1 proteins. MAb concentrations were standardized based on the assay and starting concentration is described in methods section.

Eukaryotic cell lines

Policy information about cell lines

Cell line source(s)	HEK293T cells - ATCC<br>MDCK cells - ATCC<br>A549 cells - ATCC<br>Jurkat cells expressing FcgRIIIa with NFAT-drive luciferase reporter gene - Promega, G7010<br>MDCK-SIAT1 - generated in 10.1126/science.1187816.
Authentication	Cell lines were authenticated by supplier. No other authentication at the lab level was performed.
Mycoplasma contamination	Cell lines were not tested.
Commonly misidentified lines<br>(See ICLAC register)	No commonly misidentified cell lines were used in this study.

Animals and other organisms

Policy information about studies involving animals; ARRIVE guidelines recommended for reporting animal research

Laboratory animals	6-8 week old female Balb/c mice were used for challenge studies. Mice were housed in ABSL-2 conditions with 12-hour light/dark cycles and controlled temperature and humidity.
Wild animals	No wild animals were used in this study
Field-collected samples	No field collected samples were used in this study.
Ethics oversight	Animal experiments were approved by the University of Chicago and Icahn School of Medicine at Mount Sinai IACUCs.

Note that full information on the approval of the study protocol must also be provided in the manuscript.

Human research participants

Policy information about studies involving human research participants

Population characteristics	For the 2009 MIV, 2009 pH1N1 infection, 2010 TIV, and 2014 QIV cohorts, median age was 30 years old with a range of 20-64. 63% of donors were women. Only birth date, prior influenza virus vaccination status, and sex were obtained or released to the authors for donors within these cohorts.<br><br>For the cHA vaccine trial - In Groups 1, 2, and 4 about two-thirds of the subjects were female (70.0%, 66.7%, and 62.5%, respectively), compared to 2/5 (40.0%) in Group 3 and 5/10 (50.0%) in Group 5. Most were non-Hispanic or Latino (80% to 100% per group) and black or African American (66.7% to 87.5%). The median age at enrollment ranged from 26 to 31 years across groups, with minimum age ranging from 18 to 22 and maximum age from 29 to 38. The median weight ranged from 74.7 kg to 84.2 kg and the median height ranged from 165.5 cm to 174.0 cm.<br>The most common pre-existing conditions across all treatment groups were immune system disorders (24/66, 36.4%), the majority being allergies (seasonal and food), followed by drug hypersensitivity.<br>No subjects in the placebo Groups 3 and 5 reported taking prior medications and more subjects in Group 1 (5/19, 26.3%) reported taking prior medications compared to Group 2 (1/14, 7.1%) and Group 4 (2/15, 13.3%). The proportions of subjects taking concomitant medications in Groups 1 to 5 were 84.2%, 78.6%, 100%, 60.0% and 80.0%, respectively. The most common, by therapeutic subgroup, were analgesics (20/61, 32.8%), anti-inflammatory and anti rheumatic products (19/61, 31.1%) and sex hormones and modulators of the genital system (18/61, 29.5%).

Recruitment	Participants were recruited from the local community for all cohorts. The target population reflected the community at large. All participants provided informed consent. Donors for the 2009 MIV, 2010 TIV, 2014 QIV, and 2009 pH1N1 infection had been recruited from prior studies from the local community. Participants for the cHA vaccine trial were recruited according to Bernstein et al. Lancet Infectious Disease 2020 and Nachbagauer et al. Nature Medicine 2020.The target population reflected the community at large at each of the participating study sites. Information regarding this trial was provided to potential subjects who have previously participated in vaccine trials conducted at the participating study sites.
Ethics oversight	The study protocol was approved by the IRB at the University of Chicago for all studies, the Emory University for the 2009 pH1N1 infection and 2009 MIV cohorts, and at Icahn School of Medicine at Mount Sinai, Duke University and Cincinnati Children's Hospital Medical Center (CCHMC) for the cHA vaccine study.

Note that full information on the approval of the study protocol must also be provided in the manuscript.

Clinical data

Policy information about clinical studies

All manuscripts should comply with the ICMJE guidelines for publication of clinical research and a completed CONSORT checklist must be included with all submissions.

Clinical trial registration	NCT03300050
Study protocol	Study Protocol can be accessed here - https://clinicaltrials.gov/ProvidedDocs/50/NCT03300050/Prot_000.pdf
Data collection	Gamble Program for Clinical Studies, Cincinnati Children's Hospital Medical Center 3333 Burnet Avenue, Cincinnati, OH 45229-3039<br>Duke Early Phase Clinical Research Unit, Duke Clinical Research Institute 40 Duke Medicine Circle, Durham, NC 27710<br>Studied period:<br>Date of first enrollment: 10 October 2017<br>Date of last subject completion: 09 August 2019
Outcomes	Primary Objectives<br>To assess the reactogenicity and safety through 28 days after each priming dose of cH8/1Nl LAIV (or placebo) and the booster dose of cH5/1Nl IIV +/- AS03A (or placebo) and through 28 days after each dose of IIV (cH8/1Nl IIV + AS03A and cH5/1Nl IIV + AS03A) (or placebo) in terms of rates of solicited local and general adverse events (AEs) through 7 days post-vaccination, unsolicited AEs through 28 days post vaccination, hematological and biochemical laboratory abnormalities up to Visit 13, and medically attended event (MAEs), laboratory-confirmed influenza-like illness (LC-ILi), potential immune-mediated disease<br>(plMDs), and serious adverse events (SAEs) through Visit 13.<br>Secondary Objectives<br>For this study, serum antibody titers (EC50) for the anchor and central stalk epitopes was determined. Only individuals with samples at d1, 28, 85, 113, and 420 were included in the analysis. Seroconversion was considered when titers increased by 1.5x over pre-vaccine time points (d1 or d85).

Flow Cytometry

Plots

Confirm that:

☒ The axis labels state the marker and fluorochrome used (e.g. CD4-FITC).

☒ The axis scales are clearly visible. Include numbers along axes only for bottom left plot of group (a 'group' is an analysis of identical markers).

☒ All plots are contour plots with outliers or pseudocolor plots.

☒ A numerical value for number of cells or percentage (with statistics) is provided.

Methodology

Sample preparation	HEK293T cells were transfected with plasmids to express full length membrane-bound A/California/7/2009 H1 or membrane bound mini-HA. Cells were harvested 4 days later and stained with monoclonal antibodies of interest, which were detected with an anti-human IgG-BV421.
Instrument	BD LSRFortessa
Software	Data Collection - FACSDIva<br>Anaylsis - FlowJo v10
Cell population abundance	Cell population abundance is shown for representative panels and gates are identical across individual datasets.
Gating strategy	Cells were gated on using FSC and SSC. From this gate, mAb+ cells were gated on, based on the secondary Ab only stain. Gating strategy and examples are in Supplemental Fig. 1. For mAb+ gating, histograms are represented.

☒ Tick this box to confirm that a figure exemplifying the gating strategy is provided in the Supplementary Information.
